# Mutations of Photosystem II D1 Protein That Empower Efficient Phenotypes of *Chlamydomonas reinhardtii* under Extreme Environment in Space

**DOI:** 10.1371/journal.pone.0064352

**Published:** 2013-05-14

**Authors:** Maria Teresa Giardi, Giuseppina Rea, Maya D. Lambreva, Amina Antonacci, Sandro Pastorelli, Ivo Bertalan, Udo Johanningmeier, Autar K. Mattoo

**Affiliations:** 1 Institute of Crystallography, National Research Council of Italy, CNR, Rome, Italy; 2 Institute of Plant Physiology, Martin-Luther University Halle-Wittenberg, Halle (Saale), Germany; 3 The Henry A. Wallace Beltsville Agricultural Research Center, United States Department of Agriculture, Agricultural Research Service, Sustainable Agricultural Systems Laboratory, Beltsville, Maryland, United States of America; University of Hyderabad, India

## Abstract

Space missions have enabled testing how microorganisms, animals and plants respond to extra-terrestrial, complex and hazardous environment in space. Photosynthetic organisms are thought to be relatively more prone to microgravity, weak magnetic field and cosmic radiation because oxygenic photosynthesis is intimately associated with capture and conversion of light energy into chemical energy, a process that has adapted to relatively less complex and contained environment on Earth. To study the direct effect of the space environment on the fundamental process of photosynthesis, we sent into low Earth orbit space engineered and mutated strains of the unicellular green alga, *Chlamydomonas reinhardtii,* which has been widely used as a model of photosynthetic organisms. The algal mutants contained specific amino acid substitutions in the functionally important regions of the pivotal Photosystem II (PSII) reaction centre D1 protein near the Q_B_ binding pocket and in the environment surrounding Tyr-161 (Y_Z_) electron acceptor of the oxygen-evolving complex. Using real-time measurements of PSII photochemistry, here we show that during the space flight while the control strain and two D1 mutants (A250L and V160A) were inefficient in carrying out PSII activity, two other D1 mutants, I163N and A251C, performed efficient photosynthesis, and actively re-grew upon return to Earth. Mimicking the neutron irradiation component of cosmic rays on Earth yielded similar results. Experiments with I163N and A251C D1 mutants performed on ground showed that they are better able to modulate PSII excitation pressure and have higher capacity to reoxidize the Q_A_^−^ state of the primary electron acceptor. These results highlight the contribution of D1 conformation in relation to photosynthesis and oxygen production in space.

## Introduction

The primary photochemistry leading to photosynthetic oxygen evolution and electron flow in oxygenic organisms involves a supramolecular protein-pigment complex, photosystem II (PSII) [1]–[3]. PSII is dominated by the D1–D2 reaction center protein heterodimer, which is sensitive to environmental stresses including damage from light and UV irradiation [2], [4]. Replacement of highly light-labile D1 protein is a primary event of the PSII repair cycle [5] to sustain crop productivity. It is also known that mutations or genetic substitution of some amino acids in D1 can lead to either an increase or a decrease in photosynthetic activity [6], [7].

Oxygenic photosynthesis is a phenomenon prevalent on Earth [8] while little is understood about performance of PSII in space. In space and at high altitudes, the presence of microgravity and cosmic rays, the flow of high-energy particles and ionizing radiation, dominate the environment and are hazardous, especially when the protecting atmosphere is missing [9]. Previous studies on higher plants performance in space reported an overall decrease in photosynthetic activities, mainly due to the microgravity associated lack of convection forces, resulting in alteration in the gases, water and small molecule exchange [10]–[13]. Unicellular organisms sent on a space flight underwent alterations in growth rate, developmental cycle and morphological characteristics [14], [15]. A few studies performed in low Earth orbit and on-ground facilities have focused on the impact of ionizing radiation on photosynthetic organisms [16], [17], demonstrating the negative effect of UV or gamma-radiation or fast neutrons on the light-dependent reactions and photosynthetic apparatus [16], [18]–[20]. Current state of knowledge presents space as an intrinsically complex environment, whose components interact to dramatically affect living matter while effects of individual components have been difficult to discern [21]. The importance of Earth magnetic field in maintaining photosynthetic efficiency, chlorophyll accumulation levels, and protecting PSII functionality in photoinhibitory conditions has been alluded to [22]–[24].

To gain further insights into growth and PSII performance in response to the harsh environment of space, we sent engineered and mutated strains of the unicellular green alga *Chlamydomonas reinhardtii* into low Earth orbit. This unicellular green alga is widely used as a model in studies of oxygenic photosynthesis [25]–[27] and can adapt to environmental extremes on Earth [28]. Other factors that favoured this choice included the ease in making specific amino acid substitutions in the D1 protein [6] and its ancient origin [6], [7], [27]. The amino acid substitutions in D1 were made **in** the Q_B_ binding pocket (Ala 250 and Ala 251), and close to the redox-active Tyr 161 (Val 160 and Ile 163) [1]. The Ala 251 residue, located in the Q_B_ binding pocket, was previously recognized as a key residue for D1 [6]. Earlier reports on some mutations near these regions of D1 showed a potential in acclimation of *C. reinhardtii* to radiation pressure [27] or ambient temperatures [29] on Earth. Chlorophyll *a* fluorescence induction kinetics of the strains aboard the Foton M2 spacecraft were monitored in real time using the fluorescence sensor, Photo II device [30]. Here we show that two specific D1 mutants (A251C and I163N) of *C. reinhardtii* were capable of efficient photosynthesis in the harsh environment of space. The findings are discussed in relation to the role of D1 conformation in stabilizing (and enhancing) PSII function in photosynthesis and oxygen production in space.

## Materials and Methods

### Chlamydomonas reinhardtii culture conditions

*C. reinhardtii* stock cultures were maintained at 25°C on agar plates prepared with Tris-acetate-phosphate (TAP) medium [31] under continuous illumination (∼50 µmol m^−2^ s^−1^). Liquid cultures were similarly grown but on TAP liquid medium with agitation at 150 rpm. All experiments were carried out on cell cultures in the mid-exponential growth phase.

### Production of *C. reinhardtii* D1 mutants

The intronless *C. reinhardtii* strain (IL) and its deletion mutant Del1 have been previously described [27], [32]. Del1 was transformed to create mutations in the *psbA* gene encoding the PSII reaction center protein D1. Substitutions made were: Ala 250 with Leu (A250L) and Ala 251 with Cys (A251C) – all near the Q_B_ binding pocket; Val 160 with Ala (V160A) and Ileu 163 with Asn (I163N) near the protein environment surrounding the redox-active tyrosine 161 (Y_Z_) (Figure S1). The pSH5 plasmid containing the complete intronless *psbA* gene and the 3′-flanking region was amplified in a total volume of 50 µl containing 100 ng plasmid DNA, 5 µl 10x Taq-polymerase buffer, 3 µl of 25 mM MgCl_2_, 5 µl of 2 mM dNTPs, 20 pmoles of each primer and 1 unit Taq-polymerase using Trioblock (Biometra) with the appropriate primers (Table S1). The standard PCR protocol used was: 25 cycles of 94°C denaturation (1 min), 52°C annealing (1 min), 72°C extension (2 min), with a 5-min denaturation step at 94°C in the first cycle and a 10-min extension step at 72°C in the final cycle. Amplified products were purified on agarose gels and directly used for algal biolistic transformation [33]. The point mutations were verified by gene sequencing (Seqlab, Göttingen, Germany). Only homoplasmic colonies of the *psb*A mutants, verified by standard PCR and agarose gel electrophoresis, were used.

### The Fluorescence Sensor: In-Flight monitoring of Chlorophyll a fluorescence induction kinetics

An automatic bio-device, Photo II (Figure S2), designed by Biosensor.srl (www.biosensor.it), was used to measure and store chlorophyll fluorescence induction data (also known as fluorescence transient or Kautsky effect [34]) during the space flight as well as for the control experiments performed on the ground as previously described [30]. Different strains of *C. reinhardtii* were placed in 24 measuring cells in triplicate/quadruplicate and the fluorescence measurements were recorded hourly for 20 days. The instrument allowed simultaneous determination of the following chlorophyll fluorescence parameters: F_0_, F_m_, F_v_, the ratio F_v_/F_m_ (where F_v_ = F_m_-F_0_ is the variable fluorescence), the area below the fluorescence curve, and the time to reach F_m_ in each sample. F_0_ was calculated by using an algorithm that determined the line of best fit for the initial data points recorded at the onset of illumination; the best-fit line was then extrapolated to time zero to determine F_0_ [30]. In each measuring cell, two white light LEDs were programmed to switch on to provide light (50 µmol m^−2^ s^−1^) for 7 h in a 24-h period, photoperiod necessary for the organisms to grow on Earth.

### Space flight and sample preparation

For the space flight, the algal cultures within the multicell fluorescence sensor were transported under controlled temperature 23–25±1°C to Kazakhstan cosmodrome in Baikonour. The box containing the algal strains was placed 24 hours before the launch in the UV-shielded internal part of the Biopan re-entry module of the Russian spacecraft Foton built by TsSKB-Samara (ESA Foton website). The spacecraft consisted of three modules – battery module, service module and re-entry module – of which only the latter was retrieved at landing. The Foton M2 capsule was launched in 2005, aboard a Soyuz-U rocket at a height of approximately 300 km. The mission lasted 15 days during periods of a quiet solar activity in the minimum phase of the 23^rd^ solar cycle. The total cosmic dose present inside the aircraft was 3.5±0.25 mGy, mainly due to high-energy heavy ions (Z) particles (HZE) and the secondary radiation derived from neutrons. The temperature during the flight mission and prior to entry and analysis on ground, measured by the fluorescence sensor, varied between 15–21±2°C.

Flight and ground-control samples were prepared with small volumes of high-density cell cultures that were subsequently enclosed within the multicell container (multicells) of the fluorescence sensor (Figure S2). Before sample preparation, the multicells were sterilized and filled with TAP agar (1.65%) medium. Algal cells from liquid cultures at the mid-exponential growth phase were harvested by low speed centrifugation, re-suspended in 150 µl of TAP medium to A_750nm_  = 18 and layered on the TAP agar medium in the multicells. Under sterile conditions, after cells had adsorbed (approx. 30 min), the multicells were hermetically closed and mounted in the fluorescence sensor. The experimental device was sent into space as described **above.** Following the space flight and return to Earth, the fluorescence sensor was shipped to the CNR (Rome) laboratory on day 3 after landing. The first set of multicells was immediately opened under sterile conditions and the algal strains transferred to fresh liquid TAP medium. The second set of multicells was opened on day 15 after landing and handled like the first set. In both cases, the cell cultures were re-grown in liquid medium for three days to reach a mid-exponential growth phase and then analyzed.

### Neutron irradiation and double modulation fluorescence experiments on the ground

The algal strains were irradiated with fast neutrons at a dose rate of 0.23 mSv h^−1^ for 24 h, similar to that in space, using a Super Proton Synchrotron at CERN (Conseil Européen pour la Recherche Nucléaire, Switzerland) as previously described [17]. Q_A_^−^ reoxidation kinetics were determined using a dual–modulation kinetic fluorimeter (Photon Systems Instruments, Brno) [35]. Each algal suspension (20 mg Chl mL^−1^) in TAP medium was dark-adapted for 10 min prior to each measurement. To analyze the Q_A_ reoxidation kinetics, a logarithmic regression was calculated to find the equations that best fitted the sets of data corresponding to the slope of the first phase of the fluorescence decay curve. The values were obtained by dividing the slope of the fluorescence decay curve in the first phase with the slope of the fluorescence decay relative to the IL control line. Three independent biological replicates were analyzed in triplicates.

### Excitation pressure experiment on the ground

Fluorescence quenching parameters were measured on continuously stirred and uniformly illuminated liquid cultures at 24°C by combining Fluorescence Modulated System (FMS2, Hansatech Ins., Norfolk, UK) with OxyLab instrument (Hansatech Ins., Norfolk, UK). Algal cultures in the mid-log growth phase were concentrated by centrifugation and dark-adapted for 10 min prior to the measurement. Experimental protocol [36] and the nomenclature [37] for the fluorescence analysis were the same as described. Prior to irradiation, the minimum fluorescence (F_0_) was measured by switching on the modulated light (0.004 μmol m^−2^ s^−1^); the maximum (F_m_) fluorescence was induced by a short saturating flash of 0.7 s of 9000 μmol m^−2^ s^−1^ of white actinic light. The levels of F_s_, F_m_' and F_0_' (steady state, maximum and minimum levels of Chl fluorescence for light-adapted samples, respectively) were recorded after 3-min light exposure consecutively at indicated light intensities. F_m_' was recorded during the saturating flash (0.7 s of 9000 μmol m^−2^ s^−1^) and F_0_' after the actinic light was temporarily switched off. Short-term far-red irradiation was applied to insure the oxidation of the PSII acceptor side for measuring F_0_'.

### Oxygen evolution measurements

Photosynthetic activity of the cells (20 mg mL^−1^ Chl) was measured using a Clark-type oxygen electrode (S1 electrode disk) connected to a Chlorolab 2 System and liquid-phase electrode chamber DW2/2 (Hansatech Ins., Norfolk, UK). To ensure that oxygen evolution was not limited by the carbon source available to the cells, 200 mL of a 50 mM sodium bicarbonate solution, pH 7.4, were added prior to the measurements [38]. The samples were illuminated under continuous stirring (70 *rpm*) at 24°C with increasing light intensities (from 0 to 350 µmol m^−2^ s^−1^), provided by red LED Light Source LHII/2R with a maximum at 650 nm (Hansatech Ins., Norfolk, UK). The rate of oxygen evolution was recorded continuously for 2 min. The light compensation point was calculated as the light irradiance at which net gas exchange is zero when photosynthesis and respiration rates are balanced using regression analysis of the linear part of the curve (up to 100 µmol m^−2^ s^−1^). At the end of the light exposure, the samples were dark adapted for 15 min and assayed for dark respiration.

### Protein extraction and immunoblotting

Total protein was extracted from the algal cells at the exponential growth phase (3–5×10^6^ cells mL^−1^, equivalent to 50 mg chlorophyll) by sonication on ice with a micro tip (Branson Soni®er 250, Branson Ultrasonics Corporation, Connecticut, USA) in 140 mL solution containing 30% sucrose, 5% SDS and 5% mercaptoethanol. Insoluble material was removed by centrifugation (15,000 g for 3 min) and solubilized proteins electrophoresed using discontinuous tricine/SDS-PAGE (15% acrylamide resolving gel and a 4% stacking gel) [39]. Two µg equivalent of Chl were loaded in each lane. For immunoblots, proteins separated by SDS/PAGE were electrotransferred onto nitrocellulose (BA-85, Schleicher & Schuell, Dassel, Germany) as described [40]. D1 protein was immunodecorated using the anti-*Psb*A (D1) C-terminal hen antibody (Agrisera AB, Sweden).

### RNA extraction and sqRT-PCR

*C. reinhardtii* cultures (A_750_  = 0.45, corresponding to: IL, 2.2×10^6^ cells mL^−1^; A251C, 2.5×10^6^ cells mL^−1^ and I163N, 1.2×10^6^ cells mL^−1^) were harvested by centrifugation (10 min at 4500 *rpm,* 4°C). Total RNA was isolated [41], its quality verified by electrophoresis on a 1.2% formaldehyde-agarose gel, and quantified (NanoDropTM Spectrophotometer; Thermo Scientific). DNase treatment was given using RQ1 DNase 1U µL^−1^ (Promega). The enzyme was removed by phenol/chloroform extraction. *psb*A transcripts were quantified by sqRT–PCR. RNA from each sample was reverse transcribed and amplified using the SYBR Green PCR Master Mix and MuLV Reverse Transcriptase Reagents (Applied Biosystems), following the one-step RT-PCR protocol recommended by the manufacturer. Briefly, 100 ng of total RNA in 25 µL SYBR Green PCR Master Mix 1 with 0.25 U µL^−1^ MuLV Reverse Transcriptase, 100 nM forward and reverse primers (*psb*Afor 5′ ACACTTGGGCAGACATCA 3′; *psb*Arev 5′ GGAAGTTGTGAGCGTT 3′) were subjected to the following thermal profile: 42°C for 30 min, 95°C for 10 min, 40 cycles with a denaturation step at 95°C for 15 s and an annealing/extension step at 60°C for 1 min. PCRs were performed in the PE Biosystems GeneAmp 5700 Sequence Detection System using Frosted Subskirted optical tubes and Seal Film (Microbiotech). The amplification products were heat denatured over a 35°C temperature gradient at 0.03°C s^−1^ from 60 to 95°C. A negative control without the template was run alongside to assess the overall specificity. Relative abundance of each gene was determined by the 2Δ^DDCt^ method [42]. RACK1 [43] was used as the endogenous control for calculating relative abundance. Each assay was duplicated. Primer design and their optimization in regard to primer dimerization, self-priming formation, and primer melting temperature (melting temperature of 59–60°C and product sizes between 90–150 bp) were done using Primer Quest (Integrated DNA Technologies, Coralville, IA. http://www.idtdna.com). Changes in D1 protein and *psb*A transcripts are expressed as fold-change in comparison to corresponding values of the IL ground control.

### Data analysis

Triplicated/quadruplicated biological replicates of the different *C. reinhardtii* strains were placed in the 24 multicells of the Photo II device for the space flight as well as for the on-ground experiments. All analyses were performed in biological triplicates with at least 2 technical replicates. Statistical analyses were performed using analysis of variance (one way ANOVA), and significance of differences evaluated by p-level. Differences significant at P≤0.05 are presented.

## Results

### Differential photosynthetic performance of Chlamydomonas IL parent and mutated D1 strains in low Earth orbit space Flight (LEO)

**Figure 1:**
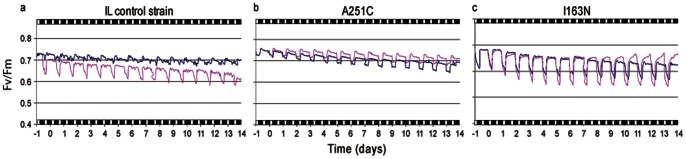
Real-time monitoring of fluorescence emission of *C. reinhardtii* strains during the Foton flight mission. The parental strain IL and its D1 mutants were sent into space inside the re-entry module of the Russian spacecraft Foton M2 capsule aboard Soyuz-U rockets launched from the Baikonur Cosmodrome in Kazakhstan. A multisensor fluorimeter specifically developed to accommodate the *Chlamydomonas* strains monitored fluorescence emission during the space flight and in ground control experiments as described in methods. The algal strains were grown in liquid TAP medium with 150 rpm agitation under a white light (50 µmol m^−2^ s^−1^) at 25°C. Cells in mid-exponential growth phase (A_750nm_  = 0.4) were harvested by low speed centrifugation, re-suspended in 150 µL of TAP medium in order to reach A_750nm_  = 18 and layered on TAP medium containing 1.65% agar. The organisms were exposed to a 17 h darkness and 7 h 50 µmol m^−2^s^−1^ daily cycle in the biocells of the fluorometer. Time zero refers to the take-off. Days 0 to14 refer to the flight period. Day -1 refers to 1 day before the take off. Pink, in-flight analysis; blue, cells analyzed on ground under simulated temperature and light conditions of the space mission.

An intronless wild type *C. reinhardtii* (IL) [44], [45] and its D1 mutants – two (A250L and A251C) near the Q_B_ binding pocket and two others (V160A and I163N) in the environment surrounding Tyr 161 (Y_Z_) [1] (Figure S1) were sent into space aboard the re-entry module of the Foton M2 spacecraft enclosed into the Photo II biodevice (Figure S2). Photosynthetic performance of each strain in flight was monitored in real time and compiled as maximum quantum yield of photosystem II (PSII) photochemical efficiency (F_v_/F_m_) (Table 1 and Figure 1). In flight, PSII photochemical efficiency of the wild type IL declined daily, recording a total decrease of 0.1 F_v_/F_m_ on the 14th (last) day of flight (Figure 1A). However, after landing (return to Earth) the F_v_/F_m_ ratio of IL, A250L and V160A strains precipitously declined (Table 1). In contrast, two mutants A251C and I163N maintained high PSII activity in flight and showed a slight increase in F_v_/F_m_ value toward the end of the flight (Figure 1B, C). The F_v_/F_m_ ratio of A251C and I163N mutants continued to remain higher even after landing, being higher than even the ground controls (Table 1).

**Table 1:** Fluorescence parameters and growth of parent strain (IL) and D1 mutants of *C. reinhardtii* after return from space.

Strains	F_0 _(% change)^a^	ΔF_v_/F_m_ (units modified)^b^	Growth^c ^(A_750Flight_/A_750Control_)
IL	+24±1	−0.200±0.004	0.4±0.07
A250L	+50±6	−0.350±0.009	n.d.
A251C	−5±2	+0.004±0.002	3.0±0.04
V160A	+48±6	−0.580±0.010	n.d.
I163N	−10±2	+0.005±0.003	1.6±0.05

The fluorescence parameters, F_0_ and F_v_/F_m,_ were recorded by the multicell fluorescence sensor during the space-flight and after landing, representing photosynthetic activity of the indicated genotypes immobilized on TAP medium and placed in the biocells of the fluorometer. Following the space-flight and landing on earth, the algal cultures were transferred to liquid TAP medium and re-growth under continuous light. The growth was determined after re-growing the space-returned genotypes in fresh liquid media for 3 days and compared to the ground controls.

a Values are a ratio of F_0_ measured one day after landing to values registered in space on the last day of the flight, using the equation: F_0_ (% change) = [(F_0_ *_after landing_*-F_0 *in flight*_)/(F_0 *in flight*_)]*100.

b Values represent the difference between F_v_/F_m_ on the day after landing and those registered in space on the last day of the flight, using the equation: ΔF_v_/F_m_ = (F_v_/F_m_ *_after landing_*- F_v_/F_m *in flight*_).

c The growth is represented as the ratio of A_750nm_ values measured after 3 days of re-growth of the space-flown samples and the corresponding ground controls. Growth  =  A_750Flight_/A_750Control_.

Differential modification of the PSII activity of the five strains in flight was also indicated by changes in the basal fluorescence level, F_0,_ whose increase signifies inactivation of PSII and decline an indication of increased non-photochemical quenching [44], [45]. The F_0_ value for the IL, A250L and V160A strains incrementally increased up to 24, 50 and 48%, respectively, while it decreased by 5–10% in the A251C and I163N mutants (Table 1). Thus, wild type IL and A250L and V160A mutants were negatively affected in space, and the A250L and V160A D1 mutants were incapable of re-growing and thus did not survive on return to Earth. In contrast, the growth of A251C and I163N D1 mutants was enhanced when re-grown on Earth upon return from space (Table 1). Because A250L and V160A mutants did not survive, the remaining analysis focused on the wild type, IL, and A251C and I163N mutants.

**Figure 2:**
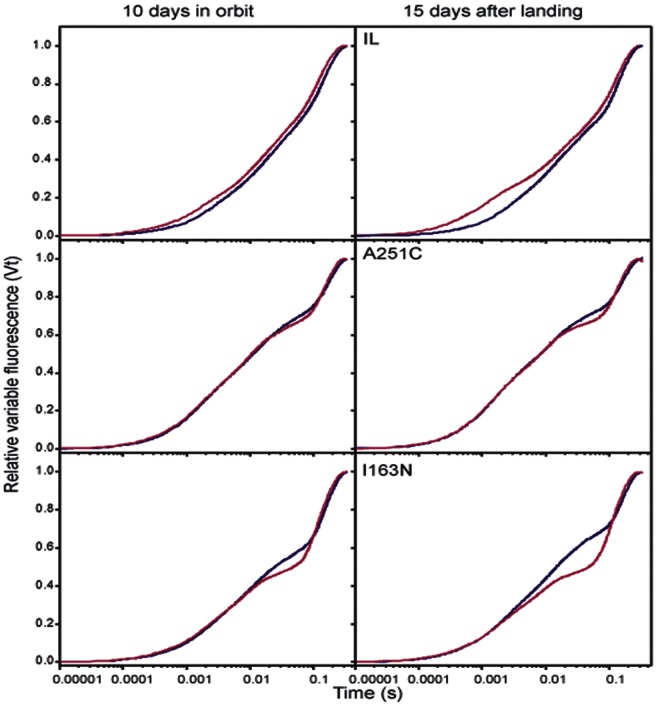
Flight-induced modifications of fluorescence transients in IL and D1-mutants (I163N and A251C) of *C. reinhardtii*. Curves in the left and right panels refer to measurements carried out in flight (10^th^ day in orbit) and 15 days after return to Earth, respectively. Fluorescence transients were measured in samples during space flight and those on ground by Photo II device. The fluorescence curves recorded at the end of the light phase are normalized for both F_0_ and F_m_ values. Curves from a representative experiment are reported – pink, in-flight analysis; blue, on-ground controls.

Space- and genotype-dependent modulation in the reduction of Q_A_ to Q_A_^−^ and the redox state of the plastoquinone (PQ) pool, as shown above by changes in F_0_ values, was further confirmed by analyzing the chlorophyll *a* fluorescence-rise kinetics, also called *OJIP* curve [45]. *OJIP* curve provides the minimal (F_0_ or *O*) and maximal (F_m_ or *P*) fluorescence levels with two intermediate peaks at about 0.002 s (step *J*) and 0.030 s (step *I*). The *O-J* rise is diagnostic of the amount of reduced Q_A_, while *J-I* and *I-P* phases denote closure of PSII reaction center and pool size of PQ and its fully reduced state [37]. The flight environment caused Q_A_^−^ accumulation in IL but not in the A251C or I163N mutant, while reduced state of PQ pool of the mutants in space was actually lower than the ground control (Figure 2). These differences were more prominent after landing and, particularly, at the end of the recovery period (15th day after landing).

### Light compensation point of A251C and I163N D1 mutants post LEO flight was higher than the parental strain

Oxygen production rates as a function of light and oxygen consumption levels in the dark of the three *Chlamydomonas* strains were measured post flight and compared to the ground controls (Table 2 and Figure S3). The rate of respiration of the IL strain was similar in the ground and post-flight samples but, as mirrored by photochemical activity, its post-flight values were significantly lower than the A251C and I163N mutants (Table 2). The light compensation point, which is a measure of light irradiance at which net gas exchange is zero when photosynthesis and respiration rates are balanced [46], [10], was calculated from the light dependency curves, and found to be remarkably higher in post-flight samples of both mutants compared to the IL strain. Environmental conditions are known to influence the compensation point of photosynthetic organisms [46].

**Table 2:** Dark respiration and light compensation point of the *C. reinhardtii* strains.

Strains	Dark respiration µmol O_2_ mg Chl^−1^ h^−1^				Light compensation point µmol photons m−2 s^−1^			
	Ground control^3a^	Ground control^15a^	Flight^3b^	Flight^15b^	Ground control^3a^	Ground control^15a^	Flight^3b^	Flight^15b^
IL	10±0.2	10±1.0	6±1.8	10±0.2	19±1.1	19±3.0	12±0.1	20±1.0
A251C	12±1.4	12±1.6	9±0.7	19±1.2	15±0.8	25±2.9	15±0.3	36±0.6
I163N	11±1.3	11±0.7	12±1.5	20±1.3	19±2.8	24±2.6	19±0.5	38±0.7

Dark respiration and light compensation point were calculated as the oxygen consumption rate in the dark and the light intensity at which oxygen consumption equals its production, respectively.

a Ground control refers to cultures handled on ground under space-simulated conditions and transferred to liquid TAP medium on day 3 (^3a^) and day 15 (^15a^) after landing.

b The algal strains following landing of the capsule were treated as described in methods.

Flight^3b^ and Flight^15b^ refer to space-sent samples after landing and which were transferred to liquid TAP medium on day 3 and day 15 after landing, respectively. Average values of one experiment in triplicate (n = 3) are shown ± SE.

### A251C and I163N D1 mutants are more efficient in reoxidizing Q_A_ than the parental strain in response to ionizing radiation exposure on the ground

**Figure 3:**
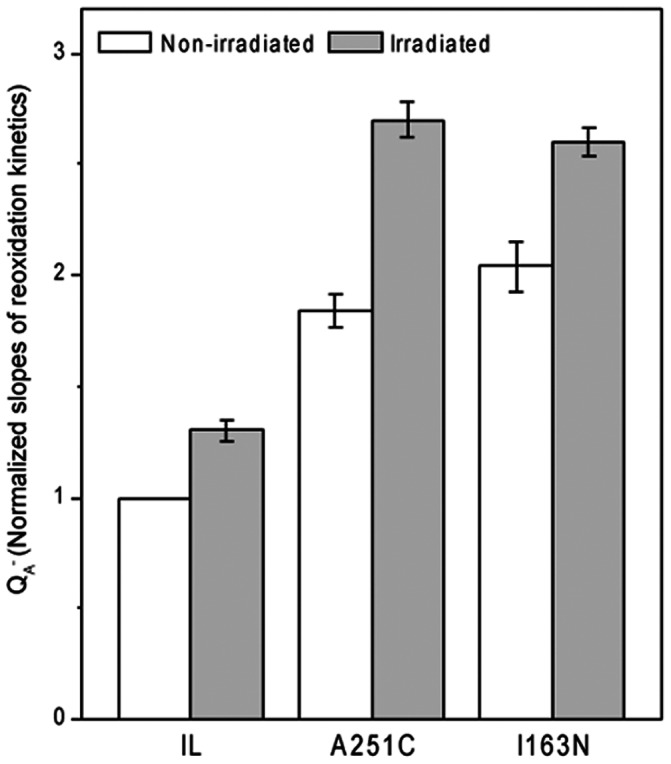
Relative Q_A_ reoxidation rates in response to on ground neutron irradiation in *C. reinhardtii* strains. To obtain the Q_A_ reoxidation kinetics slope of the first phase of the fluorescence decay curve for A251C and I163N strains, a logarithmic regression equation was used to find the best fit data sets. Then, the slope value of the fluorescence decay curve in the first 2 ms in each strain was divided by the slope value obtained for the IL control strain. The data are normalized to IL control without irradiation. Data of three independent experiments in duplicate (n = 6) ± SE are shown. The neutron dose rate given was 0.23 mSv h^−1^ for 24 h; energy varied in the range of 0–800 MeV. Measurements were performed under simulated ‘space mission’ conditions of temperature and light.

The positive effect on Q_A_ reoxidation in space in the D1 mutants compared to the parent IL strain was tested on the ground under neutron radiation. Cosmic ionizing radiation is highly complex with energies between 1 MeV and 10^3^ GeV, which can pass through the spacecraft shield, and are difficult to be mimicked on the ground [47]. Nonetheless, we exposed the three strains (IL and the two D1 mutants) to neutron irradiation at a dose of 0.23 mSv/h for 24 h, with energy of 800 MeV [27], at temperature and light conditions simulated to that in the space module. Higher order Q_A_ reoxidation kinetics was apparent in both the D1 mutants compared to the IL strain (Figure 3). Thus, mimicking only the neutron irradiation component of cosmic rays on Earth yielded similar results to those found in space-sent samples. One of the consequences of this interaction is the efficient and sustained ability to reoxidize Q_A_^−^, with a positive influence on the photosynthesis of the two D1 mutants.

### Lesser susceptibilty of A251C and I163N mutants to photoinactivation than the parental strain

**Figure 4:**
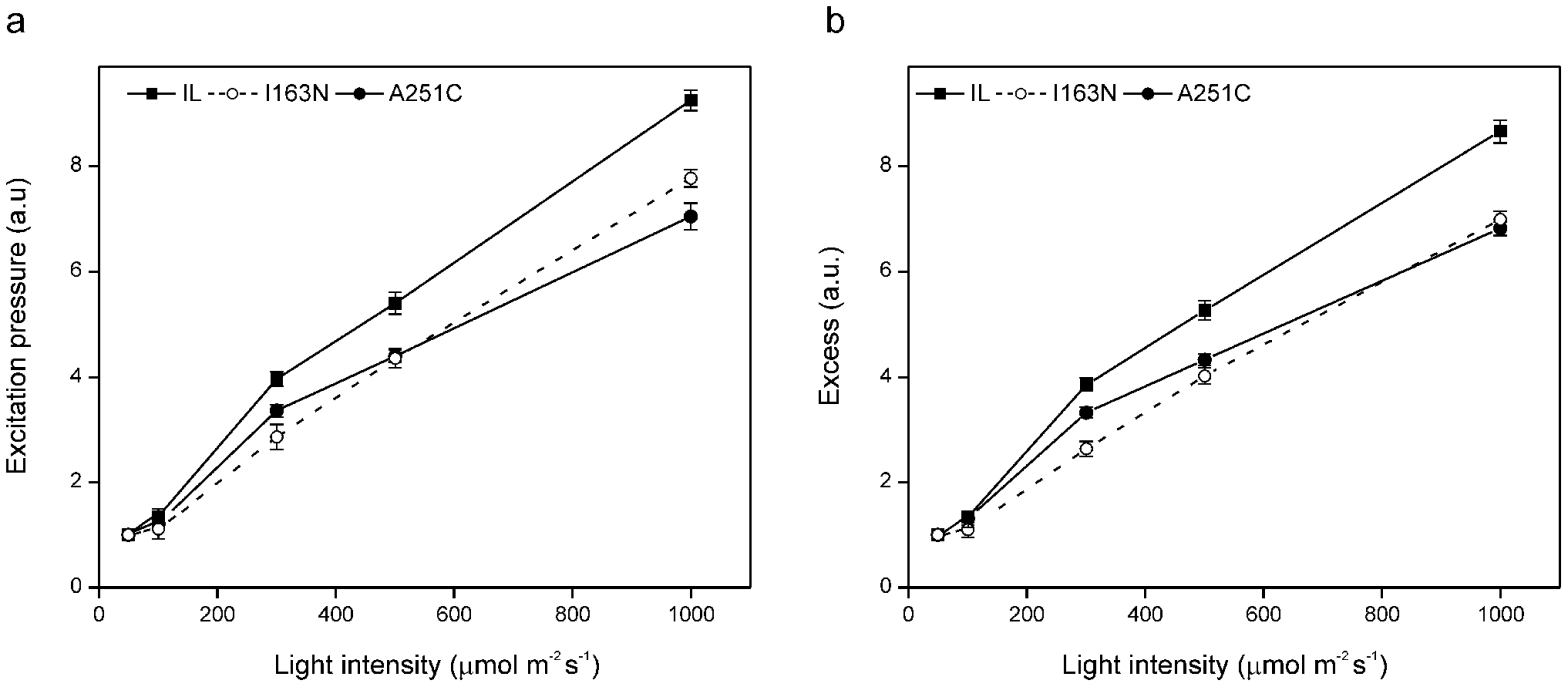
PSII sensitivity to photoinhibition of *C. reinhardtii* parent strain (IL) and D1 mutants (I163N and A251C). Parameters shown are: (**a**) Excitation pressure, 1-qP = 1-(F_m_'-F_s_)/(F_m_'-F_o_'); (**b**) *Excess,* (1-qP)(F_v_'/F_m_'), measured after 3 min exposure, at the indicated light intensities, as described [48]. Data for each strain are normalized to those obtained at the growth light intensity (50 µmol m^−2^ s^−1^). Fluorescence measurements were performed on algal cultures at mid-log growth phase containing 98±1 µg chlorophyll ml^−1^ at 24°C in continuous stirring. Average values of three independent experiments in duplicate (n = 6) are shown ± SE.

Acclimation of photosynthetic organisms, including the green algae, to photoinhibitory irradiances has been linked to their ability to modulate PSII excitation pressure (1-qP) [37], [48]. We therefore tested the excitation pressure and “excess” parameters of I163N and A251C mutants against high irradiances in comparison to the IL strain using *in vivo* fluorescence quenching analyses [37]. The parameter excitation pressure (1-qP) is a measure of the amount of light energy absorbed by closed PSII reaction centers and is based on the reduction state of the primary quinone acceptor of PSII (Q_A_) [37], [48]. At 50 μmol m^−2^ s^−1^, the 1-qP values of the A251C and I163N mutants were 0.087±0.004 and 0.071±0.006, respectively, which were slightly higher than the IL strain (0.066±0.002). As the irradiance was raised the 1-qP increased in all strains and this trend was less pronounced in the two D1 mutants. At irradiances >300 μmol m^−2^ s^−1^, the mutants showed a smaller increase in the 1-qP levels and lower accumulation of PSII reaction centers with reduced Q_A_ than the parent strain (Figure 4). The 1-qP values at 300 μmol m^−2^ s^−1^ for IL, A251C and I163N were 4.0±0.05, 3.4±0.03 and 2.9±0.23 times higher, respectively, compared to those observed at 50 μmol m^−2^ s^−1^. The parameter “excess” increased in I163N and A251C mutants as a function of light intensity but significantly slowly than in the IL strain (Figure 4). Since the parameter “excess” [E = (1-qP)(F_v_'/F_m_')] is a combined measure of the efficiency of the electron transport and non-photochemical dissipation and correlates linearly with the rate of PSII photoinactivation [48], the two D1 mutants seem less susceptible to photoinactivation than the IL strain.

### Analysis of the likely environment of D1 Ala251 and Ile163 upon mutation predicts more stable D1 in mutants

**Figure 5:**
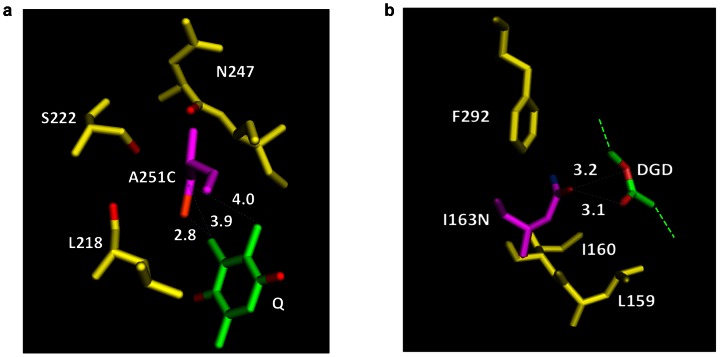
Environment of the D1 protein residue at positions 251 and 163 upon mutation to Cys and Asn, respectively*. A. Cys251 is colored in pink with the sulfur atom colored in orange. The quinone head of PQ is colored by atom type (carbon – green, oxygen – red). Residues having contact with hydrophobic Cβ atom of Ala251 are presented (yellow). Oxygen atoms of these residues that are discussed below are colored in red. Side chain placement of the mutated residue was performed with SCCOMP (19) with option to optimize positions of first-sphere residues. The Cβ atom of the residue at position 251 has two hydrophobic-hydrophobic contacts with carbon atoms of the quinone head (at 3.9 Å and 4.0 Å) in both wild type and the 3 mutants. Replacement of Ala with Cys does not cause steric problems. It provides additional stabilizing contacts (2.8 Å) to a carbon atom of the quinone head. Lengthening the marginally short distance to the quinone head can be achieved by small changes in dihedral angles. All contacts shown for this structure are stabilizing. Additional stabilization of the protein – quinone complex could result from a water mediated H-bond between the sulfur atom of Cys251 and the closest oxygen atom of the quinone head. Alternatively, stabilization of the protein structure itself could occur upon H-bond formation between the sulfur atom of Cys251 and OG atom of Ser222, assuming Cys takes a different rotamer. Either stabilizing effect could play an important role in the radiation resistance of the system. B. Asn163 is colored in pink with side chain oxygen and nitrogen atoms colored in red and blue, respectively. Residues having contact with the side chain atoms of Ile163 are presented (yellow). DGD (digalactosyl diacyl glycerol, partial representation) is colored by atom type (carbon – green; oxygen – red). Side chain placement of the mutated residue was performed with SCCOMP (19) with option to optimize positions of first-sphere residues. The side chain atoms of residue 163 have stabilizing contacts (mainly hydrophobic-hydrophobic) with Leu159 and Phe292. Replacement of Ile with Asn does not cause steric problems. It provides additional stabilizing contacts to two oxygen atoms of DGD (H-bond lengths of 3.2 and 3.1 Å). *After Sobolev V., Samish I., and Edelman M. (personal communication).

The substitutions made to defined residues, Ile163 and Ala251, in the two functionally critical pockets of the D1 protein impacted the photochemical performance of PSII in space and growth stimulation upon return to Earth. We therefore analyzed the likely environment of Ala251 and Ile163 upon mutation. Analysis of the resolved PSII structure from *Thermosynechococcus vulcanus* [1] as per previous methodology [49; Sobolev V, Samish I, and Edelman M, personal communication] shows that the hydrophobic side chain of Ala251 in D1 is in contact with the quinone head of the PQ ligand and the hydrophobic part of Leu218 (Figure 5A; Table 3). Additional contacts are formed with the backbone oxygens of Leu218, Asn247 and Ile248, as well as the side-chain oxygen atom of Ser222. Addition of a sulfur atom to the Ala moiety upon mutation to Cys (A251C) would produce a side chain that is more hydrophilic and that can form weak H-bonds with the hydrophilic environment, resulting in a stabilization of the D1 protein structure. Similar analysis shows that the hydrophobic side chain of Ile163 has productive contacts with the hydrophobic side chain of Leu159 in the D1 protein and the aromatic ring of Phe292 from the D2 protein (Figure 5B; Table 3). It also has large contact with digalactosyl diacyl glycerol (a major chloroplast membrane lipid), including contact with two of the latter's oxygen atoms. Thus, replacement of Ile163 by the more hydrophilic Asn163 would stabilize the complex, similar to the effect of the viable Cys251 mutant.

**Table 3:** List of amino acid residues in contact with mutated Cys251 and Asn163#.

Residues in contact with mutated Cys251				Residues in contact with mutated Asn163			
		Dist. Å	Surf Å^2^			Dist. Å	Surf Å^2^
218	Leu	3.1	35.4	91A	Leu*	3.7	11.8
222	Ser	3.7	15.4	159A	Leu*	3.0	19.3
247	Asn	3.0	12.8	160A	Ile*	3.4	15.1
248	Ile	3.5	10.8	162A	Pro*	1.3	75.0
250	Ala	1.3	76.1	164A	Gly*	1.3	64.7
252	His	1.3	57.5	166A	Gly	3.2	9.0
254	Tyr	3.2	7.9	288A	Cys	3.6	25.1
255	Phe	2.9	30.1	292A	Phe*	3.3	34.7
713	PQ	2.8	42.8	657A	Dgd	3.0	44.1

Distances (Dist) and surfaces (Surf) are provided. See also Figure 5a, b.

# After Sobolev V., Samish I., and Edelman M. (personal communication).

**Figure 6:**
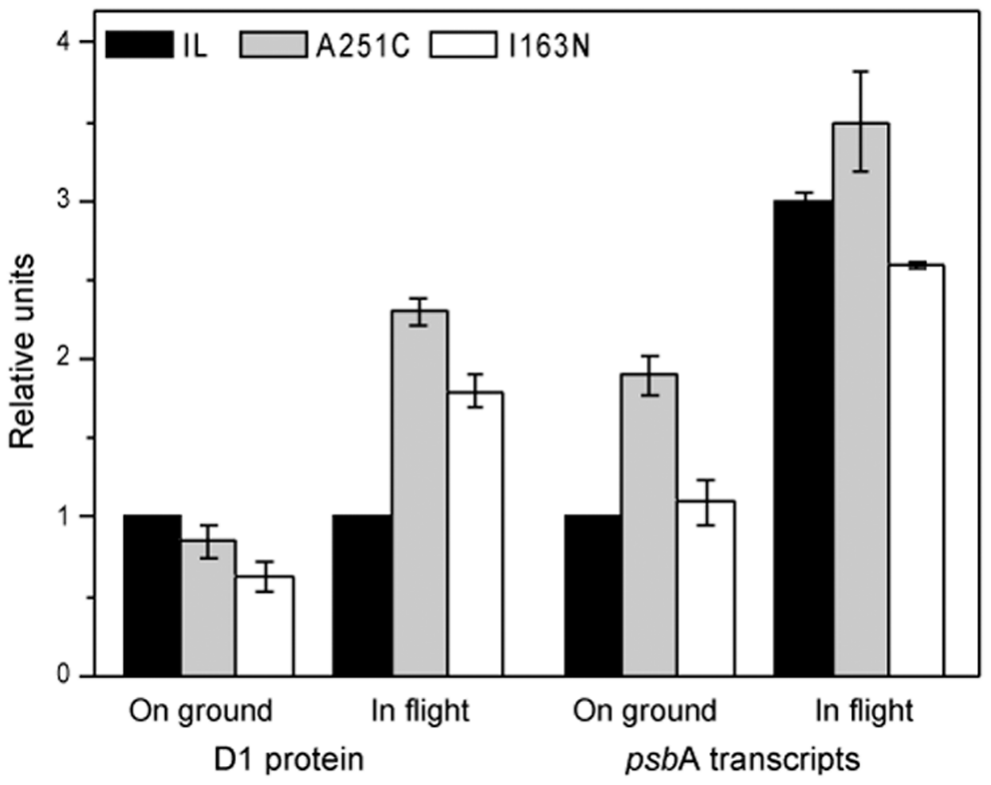
D1 content and relative abundance of *psb*A transcripts in *C. reinhardtii* strains after spaceflight. Upon return to Earth, the algal strains were transferred to liquid TAP medium for re-growth. Total protein and RNA were extracted three days later. D1 band strength was quantified by densitometric scanning of blots (n = 4). *psb*A transcripts were quantified by sqRT–PCR. Changes in D1 protein and *psb*A transcripts are expressed as fold-change in comparison to corresponding values of the IL ground control.

The above analysis predicted that the D1 protein mutated at residues Ile163 and Cys251 in *Chlamydomonas* would be less prone to degradation. This was substantiated by immunoblot data (Figure 6), which showed higher accumulation of D1 in the mutants compared to the IL strain in space-sent samples after return to Earth. In this context, the higher accumulation of D1 in the mutants correlated with higher transcription of the corresponding *psb*A gene (Figure 6). On the contrary, there was no increase in D1 protein accumulation in the IL parental strain to match its increase of *psb*A mRNA. RNA accumulation levels increased in both the mutants as well as the IL strain in space-sent samples relative to ground controls (Figure 6). These results suggest that the I163N and A251C mutations render the D1 protein relatively stable to space conditions, enabling better PSII photochemistry and better viability of the mutants in space versus the IL strain.

## Discussion

We demonstrate here that of the five strains including the control IL strain sent on a low Earth orbit space-flight, only two D1 *C. reinhardtii* mutants, I163N and A251C, efficiently carried out PSII photochemistry in space and were able to re-grow following return to Earth. Although the control IL strain survived the space flight but upon return to Earth it grew poorly. The acclimation of the I163N and A251C mutants was associated with modulation of the PSII excitation pressure (1-qP) and higher capacity to reoxidize QA−. In further evaluation of the reasons for better photochemistry of the I163N and A251C D1 mutants in space and their growth performance after return to Earth, a look at their physio-biochemical properties in comparison to the IL strain in the laboratory (on the ground) were revealing. Apart from the differences in the indicated amino acid substitutions, both D1 mutants accumulated ∼50% less Chl *a* per cell (Figure S4) and exhibited slightly higher dark respiration (Table 2), the latter result being consistent with previous data on wheat grown in space [10]. The possibly smaller absorption cross section, reflected by 50% less Chl *a* per cell, would lead to lesser light energy capture and could be a factor in promoting higher tolerance of I163N and A251C mutants to photoinhibitory light.

The I163N and A251C substitutions in the two functionally critical pockets of the D1 protein, the redox-active Tyr 161 (Y_Z_) and QB binding site (Figure S1), link the modifications to the photochemical performance of PSII and growth stimulation in space. The nature of these amino acid substitutions likely generates a more ‘resistant’ D1 protein conformation that is resilient to degradation in space. It has been shown that while there is some reduction in the rate of Q_A_^–^ reoxidation and some destabilization of the S_2/3_ states in A251C, its rate of growth and overall photosynthetic output on the ground is indistinguishable from those of wild type, possibly due to the reversibly reducible SH group of Cys exerting a positive effect on flow between the primary (Q_A_) and secondary (Q_B_) electron acceptors [6]. What remains then is the proposed increased stabilization of either the D1 protein structure or the quinine – protein complex in the Asn163 and Cys251 D1 mutants, which we speculate helps PSII to withstand the radiance rigors of space. This conclusion is supported by the non-viability of *C. reinhardtii* D1-protein mutant A250L sent up to space along with the I163N and A251C mutants. For A250L mutant, the immediate vicinity of position 251 in the protein is likely to be strongly destabilized by steric hindrance and/or hydrophobic – hydrophilic interactions (Figure 5; Table 3). As a result, it is expected that for these mutant proteins to fold and integrate into PSII, significant rearrangements in the structure of the molecule need to occur beyond the first-shell residues interacting with amino acids at positions 163 and 251. Significant structural rearrangements in the D1 protein can be expected to impinge on the photochemistry of electron transfer in the reaction center. In fact, in *Chlamydomonas* these two mutants are severely impaired in both acceptor side (Q_A_ → Q_B_) and donor side electron transfer, and show slower photoautotrophic growth under both low and high irradiance conditions [6].

Our data support an important role of D1 conformation in stabilizing (and enhancing) PSII function, and strengthen the concept of a dynamic role for D1 in mediating growth of algae and plants under normal and extreme environmental conditions [4], [5]. Future studies on developing and characterizing specific mutants of D1, which are stable and more efficient in sustaining oxygenic photosynthesis, especially under extreme environmental conditions [27], [29], are expected to have a great potential in increasing biomass. Their particular relevance falls in line with the growing demand for alternative sustainable energy, such as biofuels and food production.

## Supporting Information

Figure S1 Structure of PSII D1 (blue) and D2 (yellow) proteins depicting mutated Val160, Ile163, Ala250 and Ala251 residues, the oxygen evolving complex (OEC, orange balls) and redox active cofactors: QA and QB (pink), redox-active Tyr161 (TyrZ, red), chlorophylls (green), and non-heme Fe (red ball) (based on Umena et al., 2011 [1]).
(PDF)

Figure S2 PHOTO II automatic biodevice.
(PDF)

Figure S3 Light dependency curves of oxygen evolution of the parent strain (IL) and D1 mutants (I163N and A251C) of *C. reinhardtii*. Following the space flight and landing on earth, the algal cultures were transferred to liquid TAP medium and re-grown under continuous light for 3 days. The measurements were performed on cultures containing 20 μg Chl mL-1 at 24°C, continuous stirring and in the presence of 10 mM sodium bicarbonate. The black and white symbols correspond to samples transferred to liquid TAP medium on day 3 and day 15 after landing, respectively. Average values of one experiment in triplicate (n = 3) are shown ± SE.
(PDF)

Figure S4 Chlorophyll (Chl) *a* content per cell in IL strain and D1 mutants (I163N and A251C) of *C. reinhardtii*. Average values of three biological replicates (n = 3) are shown ± SE.
(PDF)

Table S1 List of the DNA primers used in the 2-step PCR for site-directed mutagenesis experiments. The altered nucleotides at positions 163 (I163N) and 251 (A251C) are highlighted.
(PDF)
