# Supplementary material for: Mutations of Photosystem II D1 Protein That Empower Efficient Phenotypes of Chlamydomonas reinhardtii under Extreme Environment in Space
Source: PLoS One. 2013 May 14;8(5):e64352. doi: 10.1371/journal.pone.0064352 (PMC3653854; doi:10.1371/journal.pone.0064352)
Supplement: Figure S1 — Structure of PSII D1 (blue) and D2 (yellow) proteins depicting mutated Val160, Ile163, Ala250 and Ala251 residues, the oxygen evolving complex (OEC, orange balls) and redox active cofactors: QA and QB (pink), redox-active Tyr161 (TyrZ, red), chlorophylls (green), and non-heme Fe (red ball) (based on Umena et al., 2011 [1]). (PDF) [file pone.0064352.s001.pdf]

Figure S1. Structure of PSII D1 (blue) and D2 (yellow) proteins depicting mutated Val160, Ile163, Ala250 and Ala251 residues, the oxygen evolving complex (OEC, orange balls) and redox active cofactors: QA and QB (pink), redox-active Tyr161 (TyrZ, red), chlorophylls (green), and non-heme Fe (red ball) (based on Umena et al., 2011 [1]).

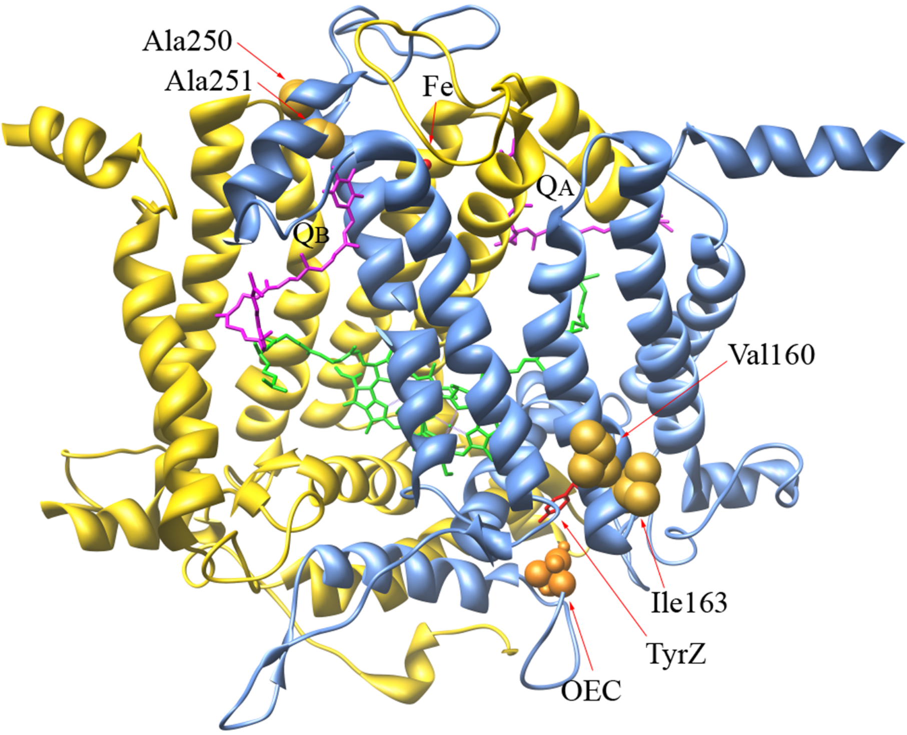
Ala250
Ala251
Fe
QA
QB
Val160
Ile163
TyrZ
OEC
