# Supplementary figures and images for: Mutations of Photosystem II D1 Protein That Empower Efficient Phenotypes of Chlamydomonas reinhardtii under Extreme Environment in Space
Source: PLoS One. 2013 May 14;8(5):e64352. doi: 10.1371/journal.pone.0064352 (PMC3653854; doi:10.1371/journal.pone.0064352)

**Figure S2** PHOTO II automatic biodevice.

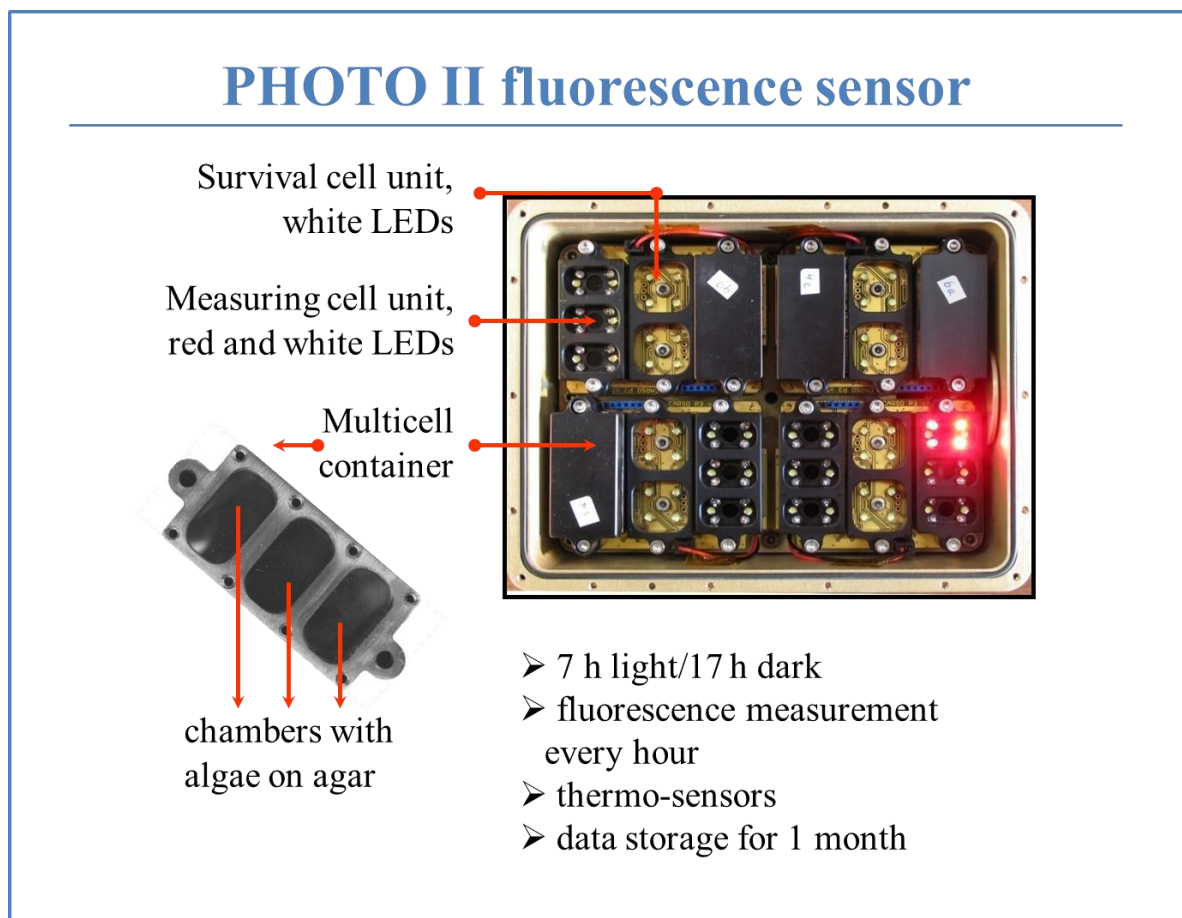

Supplement: Figure S2 — PHOTO II automatic biodevice. (PDF) [file pone.0064352.s002.pdf]
