# Supplementary material for: Mutations of Photosystem II D1 Protein That Empower Efficient Phenotypes of Chlamydomonas reinhardtii under Extreme Environment in Space
Source: PLoS One. 2013 May 14;8(5):e64352. doi: 10.1371/journal.pone.0064352 (PMC3653854; doi:10.1371/journal.pone.0064352)

Figure S3 Light dependency curves of oxygen evolution of the parent strain (IL) and D1 mutants (I163N and A251C) of *C. reinhardtii*. Following the space flight and landing on earth, the algal cultures were transferred to liquid TAP medium and re-grown under continuous light for 3 days. The measurements were performed on cultures containing 20 μg Chl mL^{-1} at 24 °C, continuous stirring and in the presence of 10 mM sodium bicarbonate. The black and white symbols correspond to samples transferred to liquid TAP medium on day 3 and day 15 after landing, respectively. Average values of one experiment in triplicate (n=3) are shown ± SE.

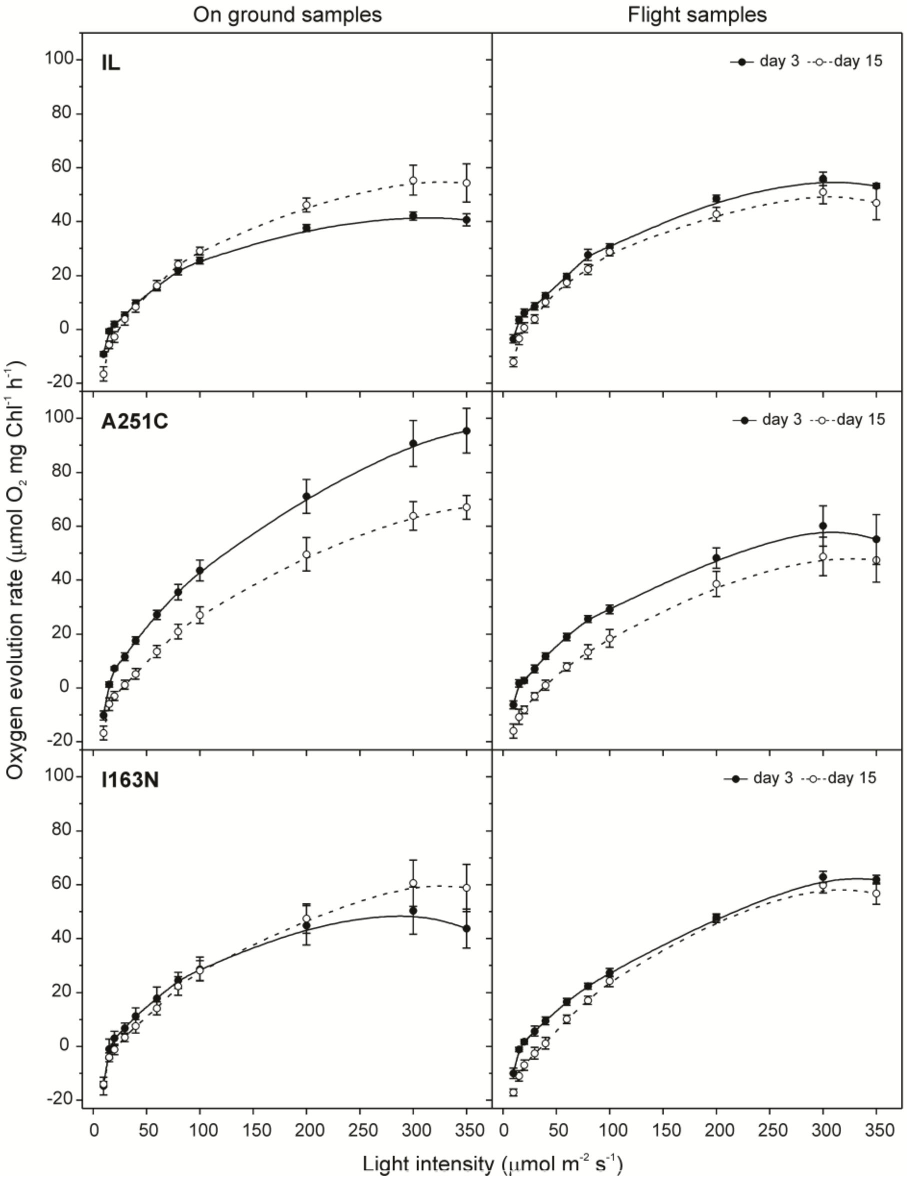

Supplement: Figure S3 — Light dependency curves of oxygen evolution of the parent strain (IL) and D1 mutants (I163N and A251C) of C. reinhardtii. Following the space flight and landing on earth, the algal cultures were transferred to liquid TAP medium and re-grown under continuous light for 3 days. The measurements were performed on cultures containing 20 μg Chl mL-1 at 24°C, continuous stirring and in the presence of 10 mM sodium bicarbonate. The black and white symbols correspond to samples transferred to liquid TAP medium on day 3 and day 15 after landing, respectively. Average values of one experiment in triplicate (n = 3) are shown ± SE. (PDF) [file pone.0064352.s003.pdf]
