# Supplementary material for: Mutations of Photosystem II D1 Protein That Empower Efficient Phenotypes of Chlamydomonas reinhardtii under Extreme Environment in Space
Source: PLoS One. 2013 May 14;8(5):e64352. doi: 10.1371/journal.pone.0064352 (PMC3653854; doi:10.1371/journal.pone.0064352)

Figure S4 Chlorophyll (Chl) *a* content per cell in IL strain and D1 mutants (I163N and A251C) of *C. reinhardtii*. Average values of three biological replicates (n=3) are shown ± SE.

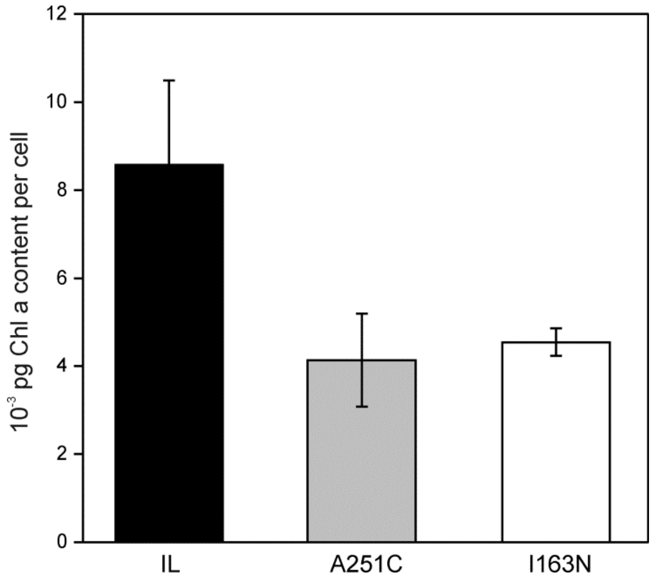

Supplement: Figure S4 — Chlorophyll (Chl) a content per cell in IL strain and D1 mutants (I163N and A251C) of C. reinhardtii. Average values of three biological replicates (n = 3) are shown ± SE. (PDF) [file pone.0064352.s004.pdf]
