# Supplementary material for: Mutations of Photosystem II D1 Protein That Empower Efficient Phenotypes of Chlamydomonas reinhardtii under Extreme Environment in Space
Source: PLoS One. 2013 May 14;8(5):e64352. doi: 10.1371/journal.pone.0064352 (PMC3653854; doi:10.1371/journal.pone.0064352)
Supplement: Table S1 — List of the DNA primers used in the 2-step PCR for site-directed mutagenesis experiments. The altered nucleotides at positions 163 (I163N) and 251 (A251C) are highlighted. (PDF) [file pone.0064352.s005.pdf]

Table S1

List of the DNA primers used in the 2-step PCR for site-directed mutagenesis experiments. The altered nucleotides at positions 163 (I163N) and 251 (A251C) are highlighted.

Step	Primers	Sequence
**1st PCR**	outer for	5’- GGTGCTGTAATCCCAACTTCT -3’
	I163N rev	5’- AGGGTAAACTAAGAATACAGC -3’
	I163N for	5’- GGCCAAGGTTCATTCTCTG -3’
	A251C rev	5’- AGCTACAATGTTGTAAGTTTCTTCTTC -3’
	A251C for	5’- CATGGTTACTTTGGTCGTCTAATC - 3’
	outer rev	5’- CTAGAGTTAGTTGAAGCTAAGTCTAGAGGGA -3’
**2nd PCR**	outer for	5’- GGTGCTGTAATCCCAACTTCT - 3’
	I163N mut-rev	5’- GAACCTTGGCC**GTT**AGGGTAAACTAAGAATACAGC -3’
	I163N mut-for	5’- CTTAGTTTACCCT**AAC**GGCCAAGGTTCATTCTCTG -3’
	A251C mut-rev	5’- GACCAAAGTAACCATG**ACA**AGCTACAATGTTGTAAG -3’
	A251C mut-for	5’- CTTACAACATTGTAGCT**TGT**CATGGTTACTTTGGTCG -3’
	outer rev	5’- CTAGAGTTAGTTGAAGCTAAGTCTAGAGGGA -3’
